# The risk of mortality from multiple primary cancers in colorectal cancer survivors: analysis of data from the South Australian Cancer Registry

**DOI:** 10.1007/s00432-025-06268-w

**Published:** 2025-07-26

**Authors:** Mulugeta Melku, Oliver G. Best, Jean M. Winter, Lauren A. Thurgood, Ganessan Kichenadasse, Molla M. Wassie, Muktar Ahmed, Erin L. Symonds

**Affiliations:** 1 Flinders Health and Medical Research Institute, College of Medicine and Public Health, Flinders University, Adelaide, SA 5042 Australia; 2 Department of Hematology and Immunohematology, School of Biomedical and Laboratory Sciences, College of Medicine and Health Science, University of Gondar, 196, Gondar, Ethiopia; 3 Medical Oncology Department, Flinders Centre for Innovation in Cancer, Flinders Medical Centre, South Adelaide Local Health Network, Adelaide, SA 5042 Australia; 4 Gastroenterology Department, Flinders Medical Centre, South Adelaide Local Health Network, Adelaide, SA 5042 Australia

**Keywords:** Colorectal cancer, Hazard ratio, Mortality, Multiple primary cancer, Standardised mortality ratio

## Abstract

**Purpose:**

Colorectal cancer (CRC) survivors face an increased risk of multiple primary cancers (MPCs), but evidence on MPC-related mortality is limited.

**Methods:**

Using data from the South Australian Cancer Registry (1982–2017), this retrospective study analysed CRC survivors diagnosed with MPCs, defined as distinct primary cancers arising ≥ 2 months after CRC diagnosis. Causes of death were categorised as index CRC, MPC, or non-cancer related. Poisson regression estimated cancer-specific mortality risk compared to the general population. Propensity score weighting was applied to balance covariate distribution between CRC survivors with and without MPC groups. A hazard ratio (HR) for all-cause mortality was estimated using a weighted dataset to assess the impact of MPC on overall survival.

**Results:**

Among 26,093 CRC survivors (181,877 person-years follow-up), the age-standardised MPC-related mortality rate was 240 per 100,000 population. Gastrointestinal, lung, haematological, and urinary tract cancers were the most common MPC-related causes of death. CRC survivors had a 45% higher risk of dying from MPCs than the general population (standardised mortality ratio = 1.45, 95%CI 1.38–1.52). Adjusted analyses showed a 58% increase in all-cause mortality among CRC survivors with MPCs (HR = 1.58, 95%CI 1.51–1.65).

**Conclusions:**

CRC survivors with MPC face significantly worse survival compared to those with a single primary CRC. Early detection and management of MPCs are essential for improving long-term survival in individuals diagnosed with CRC.

**Supplementary Information:**

The online version contains supplementary material available at 10.1007/s00432-025-06268-w.

## Introduction

Globally, colorectal cancer (CRC) is the third most diagnosed cancer and the second leading cause of cancer-related deaths. In 2020, there were 1.9 million new cases of CRC reported, and 0.9 million deaths attributed to CRC worldwide. The burden of CRC is projected to rise, with an estimated 3.2 million new cases and 1.6 million deaths in 2040 (Morgan et al. 2023), and 3.6 million new cases and 1.83 million deaths annually in 2050.

In Australia, the number of reported CRC cases increased from 6983 in 1982 to 14,396 in 2020, the lifetime risk of being diagnosed with CRC increased from 1 in 18 in 1982 to 1 in 15 in 2020. Despite this, the age-standardised rate (ASR) has declined from 58.2 to 47.4 cases per 100,000 population, and the age-standardised mortality rate (ASMR) has also decreased by almost half, from 32.4 deaths to 16.8 deaths per 100,000 population, leading to a decline in lifetime risk of dying from CRC from 1 in 32 in 1982 to 1 in 35 in 2020. Moreover, the five-year survival rate improved from 60% in 1995 to 66% in 2007, reaching 70% between 2010 and 2014 (Coleman et al. 2011), and 71% for 2016–2020. These survival improvements are attributed to enhanced CRC screening, improved treatments, and refined surgical techniques, which have collectively contributed to reduced mortality and increased survival (Marcellinaro et al. 2023).

Since CRC survival has improved, CRC survivors now face the challenge of an increased risk of multiple primary cancers (MPCs). CRC survivors have a 12–20% higher risk of developing MPCs compared to the risk of developing any cancer amongst the general population (Du et al. 2022; Melku et al. 2025; Robertson et al. 2022). This risk is even greater for long-term survivors and for those diagnosed with CRC during young adulthood (Sung et al. 2022).

Survivors diagnosed with MPC encounter further challenges related to the complexity of the disease and treatment modalities. These include increased disease burden and complications, treatment-related toxicity, weakened and dysregulated immune systems, and psychological impacts, making effective management difficult (Chitwood and Carey 2023). Despite limited data, evidence shows that MPCs are competing mortality events in CRC, accounting for 7% of the overall mortality rate, and up to 10% of all cancer-cause mortality (Dasgupta et al. 2013). There have been some attempts to elucidate the effect of MPCs on mortality estimates in cancer survivors; however, the evidence is inconclusive regarding whether the presence of an MPC increases the overall risk of mortality in CRC survivors. Ye et al. (2018) reported that while the 5-year cumulative mortality from the first cancer declined and remained stable, the cumulative mortality of the second primary cancer steadily increased in CRC survivors, particularly for long-term survivors (Ye et al. 2018). Moreover, in a competing mortality analysis using data from the Queensland Cancer Registry, Dasgupta et al. (2013) found that a secondary primary cancer reduced the hazard of both CRC-specific and non-cancer mortality while increasing the risk of mortality from other cancers (Dasgupta et al. 2013). Similarly, Guan et al. (2024) found that as survival time increased among middle-aged CRC patients, the proportion of non-cancer deaths (e.g. cardiovascular disease) and deaths from other cancers, likely including MPCs, increased, while the proportion of primary CRC-specific cancer-related deaths decreased (Guan et al. 2024). These studies suggest that MPCs are significant competing mortality events, highlighting the need for further studies to understand the contribution and impact of MPCs on overall survival.

Understanding the standardised mortality ratio (SMR) after MPC diagnosis relative to the expected cancer mortality in the general population can aid in assessing whether CRC survivors experience a higher mortality rate than expected. Furthermore, evaluating the cancer-specific SMR could help identify particular types of MPCs contributing to the increased mortality risk. However, evidence of the cancer-specific mortality risk for individuals diagnosed with an MPC is limited (Sung et al. 2022). A comprehensive understanding of both the overall and cancer-specific mortality risks could be used to refine cancer surveillance programs for CRC survivors, with the goal of reducing mortality and enhancing long-term survivorship. Therefore, this study aimed to estimate the risk of overall and cancer-specific mortality associated with diagnosis of MPC and assess the effect of MPC on all-cause mortality among CRC survivors using data from the South Australian Cancer Registry (SACR).

## Method and materials

### Data source and study design

This was a retrospective analysis of data from the SACR. The registry collates cancer-related information, including the type of cancer, date of first and subsequent primary cancer diagnoses, cancer sites and morphology. It also includes other relevant details such as place of residence, sex, usual occupation, birth and death dates, and cause of death where available. The SACR collects information on all invasive cancers but does not collect data on non-melanoma skin cancers, except for squamous cell carcinoma of the lip and the skin of the genitalia and perineum and basal cell carcinoma of the skin of the genitalia and perineum.

The primary sources of cancer information for SACR include reports from pathology laboratories, hospitals, radiology departments, and other supplementary sources such as clinicians. The SACR also obtains death data from the registry of births, deaths, and marriages, including the cause and date of death, shared through death certificates or electronic records. The SACR reviews, verifies, codes, and enters the primary cause of death as either cancer or non-cancer. If the primary cause of death is cancer, the registry codes the site and morphology based on the International Classification of Diseases for Oncology, 3rd edition (ICD-O-3) coding rules. For coding causes of death, the SACR used ICD-9 (International Classification of Diseases, 9th edition) until 2011, ICD-10 (10th edition) from 2011 to 2014, and ICD-O-3 since 2015. Available tools facilitate both automated and manual mapping and conversion between various disease coding systems and their versions, including SEER*Stat software, International Association of Cancer Registries (IARC)/International Agency for Research on Cancer (IACR) tools, and ICD-O-3/World Health Organisation conversion tools (Ferlay J et al. 2005).

### Study population

All cases with an invasive CRC with ICD-O-3 topography codes of C18 to C20 and C21.8, diagnosed between 1 January 1982 and 31 December 2017, were extracted from the SACR. To minimise the potential confounding effects of the COVID-19 pandemic-related disruptions in cancer diagnosis, treatment, and follow-up, and to ensure data consistency and quality (Canfell et al. 2023), the analysis was restricted to data from the pre-pandemic period. Individuals diagnosed with invasive CRC who met the following inclusion criteria were included: (1) survived at least two months after index CRC diagnosis, (2) had no invasive cancer prior to CRC, (3) were aged 20–89 years old at the time of index CRC diagnosis, and (4) had complete information on date of cancer diagnoses, age at diagnosis, and death date and cause of death if deceased. Cases of index CRC diagnosed as primary colorectal sarcoma, lymphoma, or leukemia and those diagnosed with synchronous cancers within two months of the index CRC diagnosis were excluded. The eligible study population were followed until 31 December 2019, at least two years from the diagnosis of index CRC.

### Ascertainment of multiple primary cancer and mortality

According to the IARC/IACR rules, MPCs are defined as the presence of histologically distinct types of invasive cancers originating in a primary site or tissue. These cancers should not be extensions, recurrences, or metastases of other primary site cancers (Report 2005). While the IARC rules for defining multiple primaries are not time-dependent, invasive cancers diagnosed within two months of the index CRC diagnosis were excluded to minimise detection bias (Ye et al. 2018). Causes of death were categorised as index CRC, MPCs, and non-cancer related. For cases where cancer was the cause of death, the primary cause of cancer-specific death was identified based on the topography and histology code of the cancer following ICD-O-3 guideline.

### Outcome measures

The outcome measures assessed in this study were the cumulative mortality and the risk of cancer-specific mortality associated with the presence of MPC compared to cancer mortality in the general population. In addition, the study assessed the effect of MPC on all-cause mortality among individuals who were first diagnosed with CRC.

### Data analysis and interpretation

As described previously(Deng et al. 2022; Gonçalves et al. 2024), for individuals who were not diagnosed with MPC, the time at risk was defined as the time in years from the diagnosis of the index CRC to death or the end of the follow-up period. Whereas for the group with MPC, the time at risk began at the diagnosis of the first MPC and continued until the date of death or end of the follow-up period, whichever came first. Participants were censored at the date of death from non-cancer causes or on December 31, 2019, whichever occurred first.

Cumulative all-cause and cause-specific mortality rates were estimated using a cumulative incidence function. The cumulative mortality from MPCs under the competing risks was estimated at 37 years after the index CRC diagnosis, with deaths from any non-MPC causes treated as competing events, as previously described (Sung et al. 2022). The indirect standardisation method was employed to standardise cancer-specific mortality rates using age, sex, and cancer-specific mortality rates from the South Australian population between 1982 and 2019 (Naing 2000). The excessive mortality risk associated with MPCs was assessed for both overall and cancer-specific mortality, reported as the SMR and the absolute excess mortality (AEM) (Sung et al. 2022). The SMRs and their 95% confidence intervals (CI) were calculated by dividing the observed number of deaths by the expected number of deaths, assuming that MPC-associated mortality follows a Poisson distribution. Similarly, AEMs and their 95% CIs were calculated as the difference between observed and expected deaths, divided by the person-years at risk and multiplied by 10,000, assuming a normal distribution of differences. SMRs and AEMs were calculated for overall MPC-associated mortality and cancer-specific mortality, and the results were stratified by sex. The proportion of deaths attributed to CRC, MPCs, and non-cancer causes was calculated by dividing the number of deaths from each cause by the total number of deaths, as previously described (Liang et al. 2025). Stata version 18 software was used to analyse the cumulative mortality rate, as well as SMRs and AEMs.

Propensity score analysis was used to ensure group comparability before estimating the effect of MPC on all-cause mortality. The propensity scores were generated based on basic demographics, calendar year of index CRC diagnosis, and location of the index cancer (colon vs rectum) to balance the distribution of covariates between individuals diagnosed with MPC and those without MPC diagnosis. The inverse probability of treatment weighting (IPTW) method was then applied to assign weights to each individual based on their likelihood of being assigned to the MPC group, given the observed covariates. The propensity scores were estimated using the “WeightIt” package in R. A Cox proportional hazards regression model was then fitted using the weighted dataset to estimate hazard ratios (HR) and 95% CI for all-cause mortality. The propensity score weights generated by the IPTW method were included in the model to account for covariate imbalances. Analysis was performed in R using the “survival” package. The proportional hazards assumption was tested using the global Schoenfeld residual test, which indicated a violation for the variable age at the diagnosis of index CRC, suggesting a time-varying effect on all-cause mortality. To address this, a Cox regression with time-varying spline analysis was fitted, treating age as a time-dependent covariate. Various spline methods, including natural spline, B-spline, penalised spline, and restricted cubic spline, were applied to check the robustness of the estimates. The estimates were consistent, and for ease of interpretation, the Cox proportional hazards model with a natural spline was applied. Knots were automatically determined using the default settings in R software and placed at the 25th, 50th, and 75th percentiles of the age distribution.

To assess the influence of different time cut-offs for defining MPC on the estimates, sensitivity analysis was conducted by calculating mortality rates, SMRs, and HRs using two distinct time intervals: two months and 6 months following the index CRC diagnosis.

## Results

### Descriptive characteristics

A total of 31,905 patients were identified from the SACR that had CRC as the index cancer. Excluding cases deceased within 2 months of index CRC diagnosis (n = 1387), unconfirmed diagnosis (n = 2880), age at CRC diagnosis < 20 or > 89 years old (n = 802), diagnosed with invasive cancer within 2 months of index CRC diagnosis (n = 564), index CRC being primary colorectal sarcoma, lymphoma or leukemias (n = 104), unknown cause of death (n = 72), and incomplete data on age of CRC diagnosis (n = 3), a total of 26,093 individuals diagnosed with CRC as their index cancer between 1982 and 2017 were included the study (Supplementary Fig. 1). The majority of index CRC cases had adenocarcinoma histology. Of these, 3836 (14.7%) were diagnosed with MPC more than two months after the diagnosis of index CRC.

The median (interquartile range (IQR)) age at the diagnosis of index CRC was 69 years (IQR: 60–77), with a majority (62.6%) being diagnosed at older ages (≥ 65 years old). Colon cancer accounted for nearly two-thirds of the index CRC, and 53% of the individuals were male (Supplementary Table 1).

### Mortality in CRC survivors

**Fig. 1:**
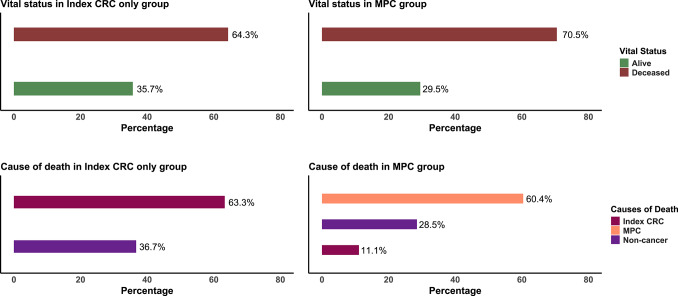
Vital status and cause of death among CRC survivors by December 31, 2019

During the follow-up time of 181,877 person-years at risk, 17,008 (65%) died. When considering the causes of death, 9350 (55%) died of the index CRC, 6024 (35.4%) died of non-cancer-related causes, and 1634 (9.6%) died of the MPC. Among those with MPC, 2705 (70.5%) died during the follow-up period, of which 1634 (60.4%) were reported to be MPC-associated deaths. In the non-MPC group, 14,303 (64.3%) died, of which 9,049 (63.3%) deaths were directly attributed to the index CRC (Fig. 1). The cumulative mortality associated with MPCs in the presence of competing deaths was found to be 7.0% (95% CI 6.6–7.3), 8.4% (95% CI 7.9–9.0), and 5.4% (95% CI 5.0–5.8) for both sexes, males, and females, respectively.

The ASMR of all MPC-associated deaths was 239.8 per 100,000 population for both sexes (ASMR = 239.8; 95%CI 228.5, 251.7). Based on the mortality rate estimates, the top five most common MPCs associated with death in male CRC survivors included gastrointestinal (GI) cancers (ASMR = 79.2, 95%CI 70.2, 89.2), lung cancer (ASMR = 67.4, 95%CI 59.3, 76.6), prostate cancer (ASMR = 35.6, 95%CI 30.5, 41.6), hematological malignancies (ASMR = 35.3, 95%CI 29.7, 42.1), and cancers of the urinary tract (ASMR = 18.5, 95%CI 14.7, 23.3). In female CRC survivors, GI cancers (ASMR = 46.5, 95%CI 40.0, 54.1), lung cancer (ASMR = 29.6, 95%CI 24.2, 36.2), breast cancer (ASMR = 28.4, 95%CI 22.8, 35.5), hematological malignancies (ASMR = 21.5, 95%CI 17.2, 26.8), and cancers of the female genital organs (ASMR = 21.0, 95%CI 16.4, 26.8) were the top five common cancer-specific causes of death. Amongst the GI cancers, oesophageal cancer (ASMR = 23.0, 95%CI 16.2, 32.7) and subsequent CRC (ASMR = 20.1, 95%CI 15.9, 25.5) were the most common causes of death in male survivors, whereas a subsequent CRC (ASMR = 18.6, 95%CI 14.7, 23.5) and pancreatic cancer (ASMR = 9.9, 95%CI 7.1, 13.7) were the most common MPCs associated with death in female survivors (Table 1).

**Table 1:** Crude and age-adjusted mortality rate of MPC-associated death among CRC survivors: Data from the South Australian cancer registry

Type of MPC	All		Males		Females	
	Crude mortality (95%CI)/100,000	ASMR (95%CI)/100,000	Crude mortality (95%CI)/100,000	ASMR (95%CI)/100,000	Crude mortality (95%CI)/100,000	ASMR (95%CI)/100,000
All cancers	898.4 (855.9, 943.0)	239.8 (228.5, 251.7)	1141.1 (1073.5, 1212.9)	292.8 (275.5, 311.2)	658.8 (608.3, 713.6)	181 (167.1, 196.1)
GI cancers*	240.8 (219.3, 264.5)	63.3 (57.6, 69.5)	296.6 (263.2, 334.3)	79.2 (70.2, 89.2)	185.7 (159.8, 215.9)	46.5 (40.0, 54.1)
CRC	75.3 (63.7, 89.1)	19.9 (16.8, 23.5)	75.3 (59.3, 95.5)	20.1 (15.9, 25.5)	75.4 (59.5, 95.4)	18.6 (14.7, 23.5)
Pancreatic cancer	57.7 (47.7, 69.9)	6 (5.0, 7.3)	76.4 (60.3, 96.7)	11.3 (8.9, 14.3)	39.3 (28.4, 54.5)	9.9 (7.1, 13.7)
Oesophageal cancer	24.7 (18.5, 33.1)	11.6 (8.7, 15.5)	34.3 (24.1, 48.8)	23.0 (16.2, 32.7)	15.3 (9.1, 25.8)	3.5 (2.1, 6.0)
Gastric cancer	33.5 (26.1, 43.1)	9 (7.0, 11.6)	48.7 (36.2, 65.4)	13.2 (9.8, 17.7)	18.6 (11.5, 29.9)	4.7 (2.9, 7.6)
Liver cancer	19.8 (14.3, 27.4)	5.7 (4.1, 7.9)	31 (21.4, 44.9)	8.9 (6.2, 12.9)	8.7 (4.4, 17.5)	2.3 (1.1, 4.5)
Gallbladder and biliary tract cancer	20.3 (14.7, 28.1)	4.9 (3.6, 6.8)	18.8 (11.7, 30.3)	4.5 (2.8, 7.2)	21.9 (14.1, 33.9)	5.4 (3.5, 8.4)
Lung cancer	180.9 (162.4, 201.5)	49.6 (44.5, 55.2)	259 (227.8, 294.4)	67.4 (59.3, 76.6)	103.8 (84.9, 126.9)	29.6 (24.2, 36.2)
Haematological malignancies	112.7 (98.3, 129.2)	28.5 (24.9, 32.7)	139.5 (117.1, 166.1)	35.3 (29.7, 42.1)	86.3 (69.2, 107.6)	21.5 (17.2, 26.8)
Prostate cancer	–	–	177.1 (151.7, 206.8)	35.6 (30.5, 41.6)	–	–
Breast cancer	–	–	–	–	–	28.4 (22.8, 35.5)
Urinary tract cancers	53.9 (44.2, 65.7)	12.9 (10.6, 15.7)	78.6 (62.3, 99.2)	18.5 (14.7, 23.3)	29.5 (20.2, 43)	6.8 (4.7, 10)
Gynaecological cancers	–	–	–	–	69.9 (54.7, 89.3)	21.0 (16.4, 26.8)
Brain cancer	25.3 (18.9, 33.8)	9.7 (7.3, 12.9)	35.4 (25, 50.1)	13 (9.2, 18.3)	15.3 (9.1, 25.8)	6.1 (3.6, 10.3)
Skin melanoma	18.1 (12.9, 25.5)	5.5 (3.9, 7.7)	26.6 (17.8, 39.6)	7.8 (5.2, 11.6)	9.8 (5.1, 18.9)	2.9 (1.5, 5.5)
Oral cavity and pharyngeal cancers	14.8 (10.2, 21.6)	4.5 (3.1, 6.5)	18.8 (11.7, 30.3)	5.5 (3.4, 8.9)	10.9 (5.9, 20.3)	2.8 (1.5, 5.1)
Unknown primary site cancers	45.1 (36.3, 56)	10 (8.1, 12.4)	46.5 (34.4, 62.9)	10.6 (7.9, 14.4)	43.7 (32.1, 59.6)	9.3 (6.8, 12.7)
All cancers excluding subsequent CRC	821.3 (780.4, 864.4)	220.6 (209.6, 232.2)	1059.7 (994.1, 1129.6)	271.8 (254.9, 289.7)	586.6 (538.6, 638.7)	164 (150.5, 178.5)
All cancers excluding prostate cancer	801.2 (760.5, 844.1)	220.7 (209.5, 232.5)	954.8 (891.2, 1022.9)	260.8 (243.5, 279.4)	658.8 (608.3, 713.6)	181 (167.1, 196.1)
All cancer excluding breast cancer	859.4 (817.5, 903.4)	227.5 (216.4, 239.2)	1141 (1073.4, 1212.8)	292.8 (275.4, 311.2)	573.9 (526.2, 625.9)	153.8 (141.1, 167.8)

All cancers: included any type of cancers diagnosed after the index CRC that fulfil the criteria of MPC; GI cancers*: included cancers of the colorectum, oesophagus, pancreas, liver, stomach, small intestine, and gallbladder and biliary tract; Haematological malignancies: included all leukemias and lymphomas. MPCs were defined as the presence of distinct primary site cancer(s) arising after 2 months of index CRC diagnosis

*ASMR* age-standardised mortality rate,*CI* confidence interval, *CRC* colorectal cancer; *GI* gastrointestinal,*MPC* multiple primary cancers.

### Risk of cancer-specific mortality from MPCs

Survivors of CRC had a 45% higher risk of dying from MPCs compared to the expected risk of cancer death in the general population (SMR = 1.45, 95%CI 1.38, 1.52), with an excess mortality rate of 27.7 cases per 10,000 population (AEM = 27.7, 95%CI 25.3, 30.2). The risk of dying from an MPC was 55% higher in males (SMR = 1.55; 95% CI 1.45, 1.64) and 27% higher in females (SMR = 1.27; 95% CI 1.17, 1.37) compared to the risk of cancer death in the general population. Except for cancers of the colorectum and unknown primary sites in males, as well as cancers of the colorectum, unknown primary sites, pancreas, brain, and skin (melanoma) in females, the risk of dying from all other specific MPC types was significantly higher in CRC survivors (Table 2 & Supplementary Fig. 2). A sensitivity analysis defining MPCs as occurring 6 months after the index CRC diagnosis demonstrated that the mortality risk from MPCs and cancer-specific mortality were consistent with those estimated using a 2-month time frame to define MPC (Supplementary Table 2).

**Table 2:** Risk of MPC-associated mortality among CRC survivors: Data from the South Australian cancer registry (1982–2017)

Type of MPC	All				Males				Females			
	Obs	Exp	SMR (95%CI)	AEM (95%CI) /10,000	Obs	Exp	SMR (95%CI)	AEM (95%CI)/10,000	Obs	Exp	SMR (95%CI)	AEM (95%CI)/10,000
All cancers	1634	1131.1	**1.45 (1.38, 1.52)**	27.7 (25.3, 30.2)	1031	667.3	**1.55 (1.45, 1.64)**	40.3 (36.3, 44.7)	603	476.3	**1.27 (1.17, 1.37)**	13.9 (11.6, 16.5)
GI cancers*	438	321.3	**1.36 (1.24, 1.50)**	6.4 (5.3, 7.7)	268	180.8	**1.48 (1.32, 1.67)**	9.6 (7.7,11.9)	170	144.7	**1.18 (1.01, 1.37)**	2.7 (1.8, 4.0)
Subsequent CRC	137	130.8	1.05 (0.89, 1.24)	0.3 (0.1, 0.7)	68	69.3	0.98 (0.77, 1.24)	-0.1 (-0.6, -0.03)	69	67.2	1.03 (0.81, 1.30)	0.3 (0.03, 0.8)
Pancreatic cancer	105	64.3	**1.63 (1.35, 1.98)**	2.3 (1.6, 2.1)	69	33	**2.1 (1.65, 2.65)**	4.0 (2.8, 5.5)	36	31.6	1.14 (0.82, 1.58**)**	0.4 (0.1, 1.1)
Oesophageal cancer	45	25.2	**1.79 (1.33, 2.39)**	1.1 (0.7, 1.7)	31	11.9	**1.77 (1.25, 2.52)**	1.4 (0.8, 2.5)	14	8.3	1.69 (1.00, 2.85)	0.7 (0.2, 1.4)
Gastric cancer	61	43.8	**1.39 (1.08, 1.79)**	0.9 (0.6, 1.5)	44	29.3	**1.50 (1.12, 2.01)**	1.7 (0.9, 2.7)	17	15.4	1.10 (0.68, 1.77)	0.2 (0.1, 0.8)
Liver cancer	36	19.6	**1.84 (1.32, 2.54)**	0.9 (0.5, 1.4)	28	13.5	**2.08 (1.44, 3.01)**	1.6 (0.9, 2.6)	8	6.7	1.19 (0.60, 2.39)	0.1 (0.03, 0.6)
Gallbladder and biliary tract cancer	37	19.5	**1.90 (1.38, 2.62)**	0.9 (0.5, 1.5)	17	8.7	**1.94 (1.21, 3.13)**	0.9 (0.4, 1.8)	20	10.7	**1.86 (1.20, 2.89)**	1.0 (0.5, 1.9)
Lung cancer	329	213	**1.54 (1.39, 1.72)**	6.4 (5.3, 7.7)	234	149.9	**1.56 (1.37, 1.77)**	9.3 (7.4, 11.5)	95	68.3	**1.39 (1.14, 1.70)**	3.0 (1.9, 4.3)
Haematological malignancies	205	123.5	**1.66 (1.45, 1.90)**	4.5 (3.5, 5.5)	126	70.2	**1.79 (1.51, 2.14)**	6.2 (4.7, 8.1)	79	54.1	**1.46 (1.17, 1.82)**	2.7 (1.8, 4.0)
Prostate cancer	–	–		–	160	101.4	**1.58 (1.35, 1.84)**	6.5 (5.0, 8.4)	–	–		–
Breast cancer	–	–		–	–	–		–	78	57.8	**1.35 (1.08, 1.68)**	2.2 (1.3, 3.4)
Urinary tract cancers	98	64.7	**1.52 (1.24, 1.85)**	1.8 (1.3, 2.6)	71	44.9	**1.58 (1.25, 2.01)**	2.9 (1.9, 4.2)	27	20.9	**1.29 (0.89. 1.88)**	0.3 (0.2, 1.4)
Gynaecological cancers	–	–		–	–	–		–	64	36.9	**1.74 (1.36, 2.22)**	4.0 (2.9, 5.6)
Brain cancer	46	23.2	**1.98 (1.48, 2.64)**	1.3 (0.8, 1.9)	32	14	**2.29 (1.62, 3,23)**	2.0 (1.2, 3.2)	14	9.5	1.47 (0.88. 2.48)	0.5 (0.1, 1.1)
Skin Melanoma	33	19.2	**1.72 (1.22, 2.41)**	0.8 (0.4, 1.3)	24	13.8	**1.73 (1.17, 2.60)**	1.1 (0.5, 2.0)	9	6	1.51 (0.79, 2.90)	0.3 (0.01, 1.0)
Oral cavity and pharyngeal cancers	27	8.2	**3.30 (2.27, 4.82)**	1.1 (0.6, 1.6)	17	7.2	**2.36 (1.47, 3.80)**	1.1 (0.5, 2.0)	10	1.4	**7.1 (3.80, 13.11)**	1.0 (0.5, 1.9)
Unknown Primary site cancers	82	79.2	1.02 (0.82, 1.26)	0.1 (0.01, 0.3)	42	37.2	1.13 (0.83, 1.53)	0.6 (0.2, 1.3)	40	42.5	0.94 (0.69, 1.28)	-0.3 (-0.9, -0.1)
All cancers excluding subsequent CRC	1470	963	**1.53 (1.45, 1.61)**	28.3 (25.9, 30.9)	941	576.3	**1.63 (1.53, 1.74)**	41.1 (37.0, 45.6)	529	397.2	**1.33 (1.22, 1.45)**	14.6 (12.3, 17.4)
All cancers excluding prostate cancer	1413	991.4	**1.43 (1.35, 1.50)**	23.9 (21.7, 26.3)	810	518.5	**1.56 (1.46, 1.67)**	34.3 (30.5, 38.5)	603	476.3	**1.27 (1.17, 1.37)**	13.9 (11.6, 16.5)
All cancers excluding breast cancer	1541	1049.6	**1.47 (1.40, 1.54)**	27.4 (25.0, 29.9)	1030	666.8	**1.55 (1.45, 1.64)**	40.2 (36.2, 44.6)	511	402.5	**1.27 (1.16, 1.39)**	12.1 (10.0, 14.6)

All cancers: included any cancers diagnosed after the index CRC that fulfil the criteria of MPC as defined in the method; GI cancers*: included cancers of the colorectum, oesophagus, pancreas, liver, stomach, small intestine, and gallbladder and biliary tract; Haematological malignancies: included all leukemias and lymphomas; Bold numeric values indicate significantly elevated risk of death beyond the expected cancer death in the general population. MPCs were defined as the presence of distinct primary site cancer(s) arising after 2 months of index CRC diagnosis

*AEM* absolute excess mortality,; *CI* confidence interval, *CRC* Colorectal cancer, *GI* Gastrointestinal; *Exp* Expected death, *MPC* Multiple primary cancers, *Obs* Observed death, *SMR* Standardised mortality ratio

### Effect of MPC on all-cause mortality in CRC survivors

CRC survivors diagnosed with MPC had a higher cumulative risk of all-cause mortality compared to those diagnosed with only an index CRC (Supplementary Fig. 3). From 1990 to 2019, the proportion of deaths attributed to MPC as a competing cause gradually increased, while the contribution of the index CRC as a cause of death declined (Supplementary Fig. 4). The hazard of all-cause mortality due to MPC increased with increasing age at CRC diagnosis (Supplementary Fig. 5). After adjusting for age (continuous), sex, calendar year of index CRC diagnosis (before and after the introduction of the National Bowel Cancer Screening Program in 2006), and location of index CRC (colon versus rectum), the presence of MPC was associated with a 58% increased risk of all-cause mortality (HR = 1.58, 95%CI 1.51, 1.65) (Table 3). The association was stronger when a 6-month cut-off period following the index CRC diagnosis was applied to define an MPC (Supplementary Table 3). Moreover, when the analysis was limited to only those individuals who survived at least 5 years after the diagnosis of the index CRC, MPC was found to be a strong predictor of all-cause mortality (HR = 4.26, 95% CI 3.99, 4.55) (Supplementary Table 4).

**Table 3:** Hazard ratio for all-cause mortality among CRC survivors based on the propensity score weighted dataset: analysis of South Australian Cancer Registry data

Characteristics		HR (95%CI)	*p* values
Sex	Male	1.25 (1.21, 1.29)	< 0.001
	Female	1.0	
Location of index CRC	Colon	1.0	
	Rectum	1.03 (1.01, 1.06)	0.04
Year of index CRC diagnosis	1982–2005	1.49 (1.44, 1.55)	< 0.001
	2006–2019	1.0	
Socioeconomic status	Lowest and low index	1.14 (1.10, 1.18)	< 0.001
	Middle index	1.05 (1.01, 1.09)	0.019
	High and highest index	1.0	
MPC	Yes	1.58 (1.51, 1.65)	< 0.001
	No	1.0	

To balance the distribution of covariates between CRC survivors with and without MPC groups, the propensity score weights for the average treatment effect (ATE) were calculated, including age, sex, socioeconomic status, year of index CRC diagnosis and location of index CRC. MPCs were defined as the presence of distinct primary site cancer(s) arising after 2 months of index CRC diagnosis. The year of index CRC diagnosis has been stratified based on the time of the implementation of the National Bowel Cancer Screening Program (NBCSP), where Australia introduced the screening program in 2006

*CI* confidence interval, *CRC* colorectal cancer, *HR* hazard ratio, *MPC* multiple primary cancer

## Discussion

In this study, the risk of dying from MPC was 45% higher in individuals diagnosed with index CRC compared to the expected cancer-related death rates in the general population. GI cancers, lung cancer, haematological malignancies, and urinary tract cancers were identified as the leading causes of MPC-related mortality. Furthermore, the presence of MPC increased the hazard of all-cause mortality by 58% in CRC survivors. Over time, the contribution of MPC to all-cause mortality gradually increased, while the contribution of index CRC declined, suggesting that subsequent primary cancers are emerging as significant competing causes of death in CRC survivors, highlighting the need for enhanced surveillance for early detection and treatment in this population.

It was found that the risk of dying from MPC was significantly elevated in CRC survivors compared to the risk of cancer death in the general population. The subsequent cancers tend to be highly proliferative and aggressive due to delays in diagnosis, making them difficult to treat effectively, potentially leading to an increased risk of death in this population (Bisht et al. 2019). In addition, the treatment options for subsequent primary cancers may be limited due to the lack of appropriate cell models for therapeutic development experiments; as a result, these cancers are often treated as solitary cancers of the affected organ or system. Due to the limited understanding of whether there are any unique biological features of MPCs and how they differ from solitary cancers, treatment effectiveness may be reduced, potentially leading to cumulative toxicity and death (Zhang et al. 2024). Evidence suggests that surveillance often focus on detecting the recurrence of the first cancer rather than identifying new primary cancers (Wilbur 2015). This can result in later-stage diagnosis of subsequent cancers when the disease is more advanced and challenging to treat effectively, ultimately increasing the risk of death in this population.

Cancer-specific mortality risk increased for most of the patients diagnosed with an index CRC and who subsequently developed MPC. While evidence remains limited, findings from a population-based study from the Surveillance, Epidemiology, and End Results (SEER) database program support this finding (Sung et al. 2022). The study reported that five-year CRC survivors diagnosed in young adulthood had a 76% higher mortality risk from subsequent primary cancers. This risk was particularly elevated for death from cancers of the brain, pancreas, small intestine, stomach, kidney, and melanoma. The physiological effects of previous cancer diagnoses and long-term sequelae of prior cancer treatments are likely to hinder the effectiveness of subsequent cancer treatments. Furthermore, the psychological and emotional impact of another cancer diagnosis can reduce survivors’ resilience, hope, treatment adherence, and ability to cope with treatment-related side effects, ultimately contributing to a lower quality of life, poor treatment outcomes and death (Andrykowski and Goedendorp 2020; Belcher et al. 2017).

Interestingly, the risk of death in CRC survivors with a subsequent primary CRC as MPC had no elevated risk of death compared to the risk of CRC-related deaths in the general population. In CRC survivors who undergo surgery for CRC resection, surveillance colonoscopy is recommended and commonly practiced for detecting recurrence, metastasis, and metachronous neoplasia. In Australia, for CRC survivors with surgical resection of CRC, the initial surveillance colonoscopy is recommended within 3–6 months for those who had an incomplete preoperative colonoscopy, and at 1 year for those who had complete preoperative colonoscopy. The findings of the initial colonoscopy guide the interval for the next colonoscopy, and generally, for those with no significant polyp, the colonoscopy will be extended to 5-year intervals. Evidence suggests that surveillance colonoscopy is effective in detecting recurrence, new polyps, and subsequent primary CRC early, allowing effective treatment and improving patient outcomes (Tjandra and Chan 2007). Surveillance colonoscopy for earlier detection of subsequent primary CRC and their precancerous lesions improves overall survival and reduces mortality due to CRC but cannot detect extra-colonic cancers. A prior study found that patients with subsequent primary CRC (i.e. both synchronous and metachronous) did not experience worse survival outcomes compared to those with only an index CRC (Barz et al. 2021). This suggests that detecting subsequent primary CRC may not significantly alter survival patterns (Yang et al. 2017), likely due to the fact the cancers are being detected earlier with colonoscopy surveillance.

While the percentage of deaths related to index CRC decreased from 1990 to 2019 in this study, the contributions of MPC and non-cancer causes to all-cause mortality showed a slight increase. This finding aligns with recently published evidence, which indicates a decline in deaths due to primary CRC and a corresponding increase in deaths from non-cancer causes and other cancers, including MPCs (Guan et al. 2024). Post-treatment surveillance in CRC patients may enable early detection of local recurrence, metastasis, subsequent polyps, and metachronous CRC at the early stage (Snyder et al. 2018), likely reducing CRC-specific mortality. This results in a high proportion of deaths from extra-colonic MPCs and non-cancer as a competing event of overall mortality. In addition, improvements in CRC therapies over the years could contribute to the low rate of CRC-related deaths while enabling survivors to live longer, giving them additional years during which MPC may develop. The implementation of cancer surveillance programs for common cancer and clinical cancer registries partly contributes to the improvement of the detection and reporting of cancer and associated mortality (Roder et al. 2015). These programs also help improve data quality and completeness by linking cancer records with vital statistics, enabling reliable attribution of deaths to cancers, including MPCs.

After adjusting for other covariates, diagnosis of an MPC was associated with a 58% higher risk of all-cause mortality compared to those without MPC. The association strengthened when a 6-month cut-off period following the index CRC diagnosis was used to define MPC, and when the study was limited to participants who survived at least 5 years after the index CRC diagnosis. This indicates that the time frame for determining when a cancer is defined as an MPC does not alter the direction or significance of risk association with all-cause mortality. This suggests that MPC is a significant predictor of all-cause mortality, particularly in long-term survivors. Although a direct comparison with published literature is challenging due to methodological differences, the findings from Dasgupta et al. (2013) align with our results. They reported that a second primary cancer diagnosis led to a significant reduction in CRC- and non-cancer-related mortality, while simultaneously increasing the risk of death from other cancers (Dasgupta et al. 2013). Similarly, Jia et al. (2020) found that CRC patients with subsequent primary cancers had notably poorer survival outcomes compared to those with CRC alone, indicating that MPC contributes to a significantly higher mortality risk (Jia et al. 2020). These studies reinforce the notion that the development of MPCs in CRC survivors leads to worse overall survival, supporting the observed higher hazard ratio for all-cause mortality in this population.

This study’s key strength lies in the use of a large state-wide cancer registry, which included South Australian population data from cancer survivors over a follow-up period of more than three decades, providing valuable insights into the long-term survival outcomes and implications of MPCs among CRC survivors. This information is essential for tracking the progress in reducing mortality among CRC survivors over time. Furthermore, this study assessed the risks of dying from MPCs of specific cancer types, supporting informed decisions about different cancer surveillance requirements. This highlights the need to expand post-CRC surveillance to other key cancer types, in addition to detecting early recurrences, metastasis, and new primary CRCs. In estimating the impact of MPC on all-cause mortality, the application of propensity score weighting techniques was another strength, as it minimises potential bias by balancing baseline characteristics between CRC survivors with and those without MPCs.

Limitations of this study included the lack of clinical and treatment information such as cancer stage, grade, tumour progression, treatment type and treatment response. This restricted the ability to further stratify the analysis based on such variables that might influence survival outcomes. Moreover, the lack of such detailed information restricted the development of the propensity score to include only basic demographics and the initial CRC location, which may limit the generalisability of our findings on the impact of MPC on all-cause mortality risk. Furthermore, although propensity score weighting was applied to balance covariates, the possibility of residual confounding from lifestyle and environmental factors may not be excluded. Despite these limitations, this study makes a significant contribution by providing valuable insights into the long-term survival risk of MPCs, which can inform future research and clinical practice aimed at improving outcomes for cancer survivors.

## Conclusions

In conclusion, CRC survivors faced an elevated risk of mortality from subsequent primary cancers, with MPCs emerging as a strong predictor of all-cause mortality and one of the most common competing causes of death. Furthermore, the cumulative hazard of all-cause mortality increases over time among CRC survivors who developed MPCs, suggesting that as survivors live longer, the risk of developing MPCs becomes more pronounced, and thereby increasing the risk of death. These findings highlight the need for enhanced cancer surveillance, particularly focusing on the early detection of extracolonic MPCs for long-term CRC survivors. Designing and implementing targeted follow-up strategies for extra-colonic cancer surveillance in CRC survivors could lead to improved survival rates and reduce mortality in this high-risk group. In addition, future research should consider stratifying the analyses by tumour characteristics, such as stage, degree of differentiation, and histological subtype, as this may provide a better understanding and deeper insight into risk stratification and the clinical implications.

## Electronic supplementary material

Below is the link to the electronic supplementary material.

Supplementary Material 1.

## Data Availability

The data used in this study were obtained from the South Australian Cancer Registry (SACR). The authors accessed the data under a data use agreement with SACR. The data that supports the findings of this study are available in the manuscript and supplementary files. Upon request, additional information can be obtained from the corresponding authors.
