# Supplementary material for: The risk of mortality from multiple primary cancers in colorectal cancer survivors: analysis of data from the South Australian Cancer Registry
Source: J Cancer Res Clin Oncol. 2025 Jul 26;151(7):222. doi: 10.1007/s00432-025-06268-w (PMC12297194; doi:10.1007/s00432-025-06268-w)
Supplement: Supplementary file 1 — Supplementary Material 1. [file 432_2025_6268_MOESM1_ESM.docx]

**The risk of mortality from multiple primary cancers in colorectal cancer survivors: Analysis of data from the South Australian Cancer Registry**

Mulugeta Melku^1,2^, Oliver G Best^1^, Jean M Winter^1^, Lauren A Thurgood^1^, Ganessan Kichenadasse^1,3^, Molla M Wassie^1^, Muktar Ahmed^1^, Erin L Symonds^1,4^

^1^Flinders Health and Medical Research Institute, College of Medicine and Public Health, Flinders University, South Australia, Australia, 5042

^2^Department of Hematology and Immunohematology, School of Biomedical and Laboratory Sciences, College of Medicine and Health Science, University of Gondar, Ethiopia, 196

^3^Medical Oncology Department, Flinders Centre for Innovation in Cancer, Flinders Medical Centre, South Adelaide Local Health Network, Bedford Park, South Australia, Australia, 5042

^4^Gastroenterology Department, Flinders Medical Centre, South Adelaide Local Health Network, Bedford Park, South Australia, Australia, 5042

**Corresponding authors**

Mulugeta Melku: [gobe0011@flinders.edu.au](mailto:gobe0011@flinders.edu.au).

**Co-authors’ email**

OGB: [giles.best@flinders.edu.au](mailto:giles.best@flinders.edu.au).

JMW: [jean.winter@flinders.edu.au](mailto:jean.winter@flinders.edu.au).

LAT: [lauren.thurgood@flinders.edu.au](mailto:lauren.thurgood@flinders.edu.au).

GK: [ganessan.kichenadasse@flinders.edu.au](mailto:ganessan.kichenadasse@flinders.edu.au).

MMW: [molla.wassie@flinders.edu.au](mailto:molla.wassie@flinders.edu.au).

MA: [muktar.ahmed@flinders.edu.au](mailto:muktar.ahmed@flinders.edu.au).

ELS: [Erin.Symonds@sa.gov.au](mailto:Erin.Symonds@sa.gov.au).

**Supplementary Tables**

**Supplementary Table 1**: Characteristics of cases with an index colorectal cancer diagnosis: Data from the South Australian cancer registry (1982-2017).

| **Variable** |  | **Frequency** | **Percent** |
| --- | --- | --- | --- |
| **Sex** | Male | 13,880 | 53.2 |
|  | Female | 12,213 | 46.8 |
| **Age at CRC diagnosis, year** | 20-49 | 2,228 | 8.5 |
|  | 50-64 | 7,544 | 28.9 |
|  | ≥ 65 | 16,321 | 62.6 |
| **Location of CRC** | Right-sided colon | 8,996 | 34.5 |
|  | Left-sided colon | 7,754 | 29.7 |
|  | Rectum | 9,343 | 35.8 |
| **Socio-economic status (quintile)** | Lowest | 5,092 | 19.5 |
|  | Low | 5,200 | 19.9 |
|  | Middle | 5,564 | 21.3 |
|  | High | 5,022 | 19.3 |
|  | Highest | 5,215 | 20 |

CRC: Colorectal cancer; Socioeconomic status is the socioeconomic index for areas reported by the Australian Bureau of Statistics (ABS), which ranks areas into quintiles based on their relative socioeconomic advantages and disadvantages.

**Supplementary Table 2:** Risk of MPC-associated mortality among CRC survivors using 6 months as a cut-off time to define MPC: Data from the South Australian cancer registry

| **Type of MPC** | **All** | | | | **Males** | | | | **Females** | | | |
| --- | --- | --- | --- | --- | --- | --- | --- | --- | --- | --- | --- | --- |
|  | **Obs** | **Exp** | **SMR (95%CI)** | **AEM (95%CI) /10,000** | **Obs** | **Exp** | **SMR (95%CI)** | **AEM (95%CI)/10,000** | **Obs** | **Exp** | **SMR (95%CI)** | **AEM (95%CI)/10,000** |
| All cancers | 1,583 | 1,070 | **1.48 (1.41, 1.55)** | 30.3 (27.8, 33.0) | 998 | 629 | **1.59 (1.49, 1.69)** | 43.9 (39.5, 48.6) | 585 | 452.3 | **1.29 (1.19, 1.40)** | 15.5 (13.0, 18.4) |
| GI cancers* | 426 | 304 | **1.40 (1.27, 1.54)** | 7.2 (6.0, 8.6) | 262 | 170.2 | **1.54 (1.36, 1.74)** | 10.9 (8.8, 13.4) | 164 | 138 | **1.19 (1.02, 1.39)** | 3.0 (2.0, 4.4) |
| CRC | 134 | 124 | 1.08 (0.91, 1.28) | 0.6 (0.3, 1.1) | 66 | 65.3 | 1.01 (0.79, 1.29) | 0.1 (0.03, 0.7) | 68 | 64.1 | 1.06 (0.84, 1.25) | 0.5 (0.1, 1.2) |
| Pancreatic cancer | 102 | 60.8 | **1.68 (1.38, 2.04)** | 2.4 (1.7, 3.3) | 67 | 31.1 | **2.15(1.70, 2.74)** | 4.3 (3.0, 5.9) | 35 | 30 | 1.17 (0.84, 1.62) | 0.6 (0.2, 1.4) |
| Oesophageal cancer | 42 | 23.8 | **1.76 (1.30, 2.39)** | 0.3 (0.6, 1.7) | 29 | 16.5 | **1.76 (1.22, 2.53)** | 1.4 (0.8, 2.5) | 13 | 8 | 1.65 (0.96, 2.84) | 0.6 (0.2, 1.4) |
| Gastric cancer | 61 | 41.4 | **1.47 (1.15, 1.90)** | 1.2 (0.7, 1.8) | 44 | 27.6 | **2.27 (1.69, 3.06)** | 1.9 (1.1, 3.1) | 17 | 14.7 | 1.16 (0.72, 1.86) | 0.2 (0.03, 0.8) |
| Liver cancer | 35 | 18.5 | **1.89 (1.36, 2.63)** | 0.9 (0.5, 1.5) | 28 | 12.7 | **2.21 (1.53, 3.21)** | 1.8 (1.0, 2.9) | 7 | 6.3 | 1.10 (0.53, 2.31) | 0.1 (0.03, 0.7) |
| Gallbladder and biliary tract cancer | 35 | 18.5 | **1.90 (1.36, 2.64)** | 0.9 (0.5, 1.5) | 17 | 8.3 | **2.06 (1.28, 3.31)** | 1.1 (0.5, 2.0) | 18 | 10.2 | **1.76 (1.11, 2.80)** | 0.9 (0.4, 1.8) |
| Lung cancer | 320 | 201 | **1.59 (1.43, 1.78)** | 7.0 (5.8, 8.4) | 226 | 141 | **1.60 (1.41, 1.83)** | 10.1 (8.1, 12.5) | 94 | 64.6 | **1.45 (1.19, 1.78)** | 3.4 (2.3,4.9) |
| Haematological malignancies | 202 | 117 | **1.73 (1.50, 1.98)** | 5.0 (4.0, 6.2) | 123 | 66.3 | **1.86 (1.56, 2.21)** | 6.8 (5.1, 8.8) | 79 | 51.5 | **1.54 (1.23, 1.91)** | 3.2 (2.1, 4.6) |
| Prostate cancer | __ | __ |  | __ | 156 | 96.3 | **1.62 (1.39, 1.90)** | 7.1 (5.5, 9.2) | __ | __ | __ | __ |
| Breast cancer | __ | __ |  | __ | __ | __ |  | __ | 72 | 54.8 | **1.31 (1.04. 1.65** | 2.0 (1.2, 3.2) |
| Urinary tract cancers | 93 | 61.3 | **1.52 (1.24, 1.86)** | 1.9 (1.3, 2.7) | 66 | 42.4 | **1,56 (1.22, 1.98)** | 2.6 (1.6, 4.0) | 27 | 19.9 | 1.36 (0.93, 1.97) | 0.8 (0.3, 1.7) |
| Gynaecologic cancers | __ | __ |  | __ | __ | __ |  | __ | 61 | 35 | **1.74 (1.36, 2.24)** | 3.0 (2.0, 4.4) |
| Brain cancer | 45 | 21.8 | **2.07 (1.54, 2.77)** | 1.4 (0.9, 2.0) | 31 | 13.1 | **2.37 (1.67, 3.37)** | 2.1 (1.3, 3.4) | 14 | 9 | 1.57 (0.92, 2.63) | 0.6 (0.2, 1.4) |
| Skin Melanoma | 31 | 18.2 | **1.71 (1.20, 2.43)** | 0.8 (0.4, 1.3) | 23 | 13 | **1.77 (1.18, 2.67)** | 1.2 (0.6, 2.2) | 8 | 5.7 | 1.41 (0.71, 2.82) | 0.2 (0.03, 0.8) |
| Oral cavity and pharyngeal cancer | 26 | 7.7 | **3.38 (2.30, 4.97)** | 1.1 (0.6, 1.7) | 16 | 6.8 | **2.37 (1.45, 3.87)** | 1.1 (0.5, 2.0) | 10 | 1.4 | **7.41 (3.99, 13.77)** | 1.0 (0.5, 2.0) |
| Unknown Primary site cancers | 77 | 75 | 1.03 (0.82, 1.28) | 0.1 (0.01, 0.4) | 38 | 35 | 1.09 (0.79, 1.49) | 0.4 (0.1, 1.0) | 39 | 40.4 | 0.97 (0.71, 1.32 | -0.1 (-0.7, -0.03) |
| All cancers excluding subsequent CRC | 1426 | 911 | **1.57 (1.49, 1.65)** | 30.8 (28.2, 33.6) | 913 | 543.6 | **1.68 (1.57, 1.79)** | 44.6 (40.2, 49.4) | 513 | 377 | **1.36 (1.25, 1.48)** | 16.1 (13.5, 19.0) |
| All cancers excluding prostate cancer | 1369 | 937.3 | **1.46 (1.39, 1.54)** | 26.2 (23.8, 28.8) | 784 | 488 | **1.61 (1.50, 1.72)** | 54.7 (49.6, 60.1) | 585 | 452.3 | **1.29 (1.19, 1.40)** | 15.5 (13.0, 18.4) |
| All cancers excluding breast cancer | 1496 | 993 | **1.51 (1.43, 1.59)** | 30.0 (27.5, 32.8) | 997 | 628.7 | **1.59 (1.49, 1.69)** | 43.8 (39.4, 48.5) | 499 | 382 | **1.31 (1.20, 1.43)** | 14.0 (11.6, 16.8) |

AEM: Absolute excess mortality; CI: confidence interval; CRC: Colorectal cancer; GI: Gastrointestinal; Exp: Expected death; MPC: Multiple primary cancers; Obs: Observed death; SMR: Standardised mortality ratio. All cancers: included any type of cancers diagnosed after 6 months after the index CRC diagnosis that fulfil the criteria of MPC; GI cancers*: included cancers of the colorectum, oesophagus, pancreas, liver, stomach, small intestine, and gallbladder and biliary tract; Haematological malignancies: included all leukemias and lymphomas; Bold numeric values indicate significantly elevated risk of death beyond the expected cancer death in the general population. For results included in this table, the analysis was limited to study participants who survived at least 6 months after being diagnosed with index CRC.

**Supplementary Table 3: Hazard ratio for all-cause mortality among CRC survivors based on the propensity score weighted dataset using 6-months as a cut-off time to define MPC: analysis of South Australian Cancer Registry data**

| **Characteristics** |  | **HR (95%CI)** | **p-values** |
| --- | --- | --- | --- |
| **Sex** | Male | 1.26 (1.22, 1.30) | <0.001 |
|  | Female | 1.0 |  |
| **Location of index CRC** | Conon | 1.0 |  |
|  | Rectum | 1.06 (1.02, 1.09) | <0.001 |
| **Year of index CRC diagnosis** | 1982-2005 | 1.41 (1.36, 1.46) | <0.001 |
|  | 2006-2019 | 1.0 |  |
| **Socioeconomic status** | Lowest and low index | 1.14 (1.10, 1.18) | <0.001 |
|  | Middle index | 1.05 (1.01, 1.09) | 0.029 |
|  | High and highest index | 1.0 |  |
| **MPC** | Yes | 1.96 (1.87, 2.06) | <0.001 |
|  | No | 1.0 |  |

CI: Confidence interval; CRC: Colorectal cancer; HR: hazard ratio; MPC: Multiple primary cancer. MPC was defined as the presence of distinct primary site cancer(s) arising after 6 months of index CRC diagnosis. To balance the distribution of covariates between CRC survivors with and without MPC groups, the propensity score weights for the average treatment effect (ATE) were calculated, including age, sex, socioeconomic status, year of index CRC diagnosis and location of index CRC. The year of index CRC diagnosis has been classified based on the time of the implementation of the National Bowel Cancer Screening Program (NBCSP), where Australia introduced the screening program in 2006. For results included in this table, the analysis was limited to study participants who survived at least 6 months after being diagnosed with index CRC.

**Supplementary Table 4: Hazard ratio for All-cause mortality among individuals diagnosed with CRC who survived at least 5 years, based on the propensity score weighted dataset, using 2 months as a cut-off time to define MPC: analysis of South Australian Cancer Registry data**

| **Characteristics** |  | **HR (95%CI)** | **p-values** |
| --- | --- | --- | --- |
| **Sex** | Male | 1.29 (1.23, 1.36) | <0.001 |
|  | Female | 1.0 |  |
| **Location of index CRC** | Colon | 1.0 |  |
|  | Rectum | 1.09 (1.03, 1.14) | <0.001 |
| **Year of index CRC diagnosis** | 1982-2005 | 1.62 (1.51, 1.73) | <0.001 |
|  | 2006-2019 | 1.0 |  |
| **Socioeconomic status** | Lowest and low index | 1.13 (1.07, 1.19) | <0.001 |
|  | Middle index | 1.09 (1.02, 1.16) | 0.007 |
|  | High and highest index | 1.0 |  |
| **MPC** | Yes | 4.26 (3.99, 4.55) | <0.001 |
|  | No | 1.0 |  |

CI: Confidence interval; CRC: Colorectal cancer; HR: hazard ratio; MPC: Multiple primary cancer. To balance the distribution of covariates between CRC survivors with and without MPC groups, the propensity score weights for the average treatment effect (ATE) were calculated, including age, sex, socioeconomic status, year of index CRC diagnosis and location of index CRC. For results included in this table, the analysis was limited to study participants who survived at least 5 years after being diagnosed with index CRC. The year of index CRC diagnosis has been classified based on the time of the implementation of the National Bowel Cancer Screening Program (NBCSP), where Australia introduced the screening program in 2006.

**Supplementary Figures**

Cases diagnosed with CRC from 1982-2017 (N = 36,402)

Excluded (n = 10,309)

- Prior invasive cancer (n = 4,497)
- Unconfirmed diagnosis of index CRC (n = 2,880)
- Deceased within 2 months of index CRC diagnosis (n = 1,387)
- Cases diagnosed with synchronous MPC within 2 months of index CRC (n = 564)
- Age at index CRC diagnosis <20 or >89 (n = 802)
- Primary colorectal lymphoma/leukemia/sarcoma (n = 104)
- Unknow cause of death (n=72)
- Unknown age at index CRC diagnosis (n = 3)

Included (n = 26,093)

**Population**

**Exclusion**

**Inclusion**

**Supplementary Figure 1**: Study participant eligibility and selection flow chart


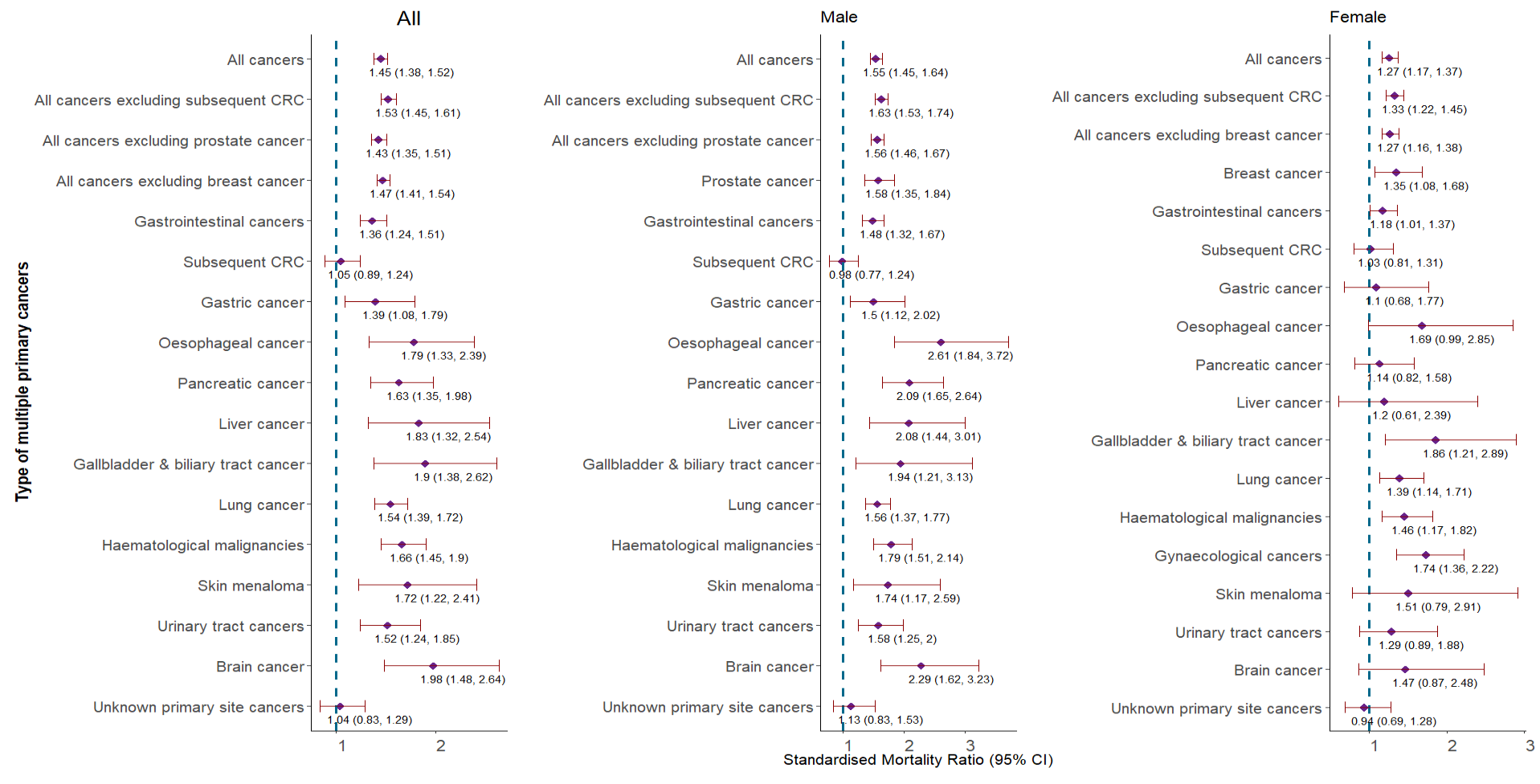


**Supplementary Figure 2:** Risk of MPC-associated mortality among CRC survivors: Data from the South Australian cancer registry: 1982-2017

**Supplementary Figure 3:** Nelson-Aelen cumulative hazard of all-cause mortality comparing groups with index CRC only and MPC. CRC: Colorectal Cancer; MPC: Multiple Primary cancer


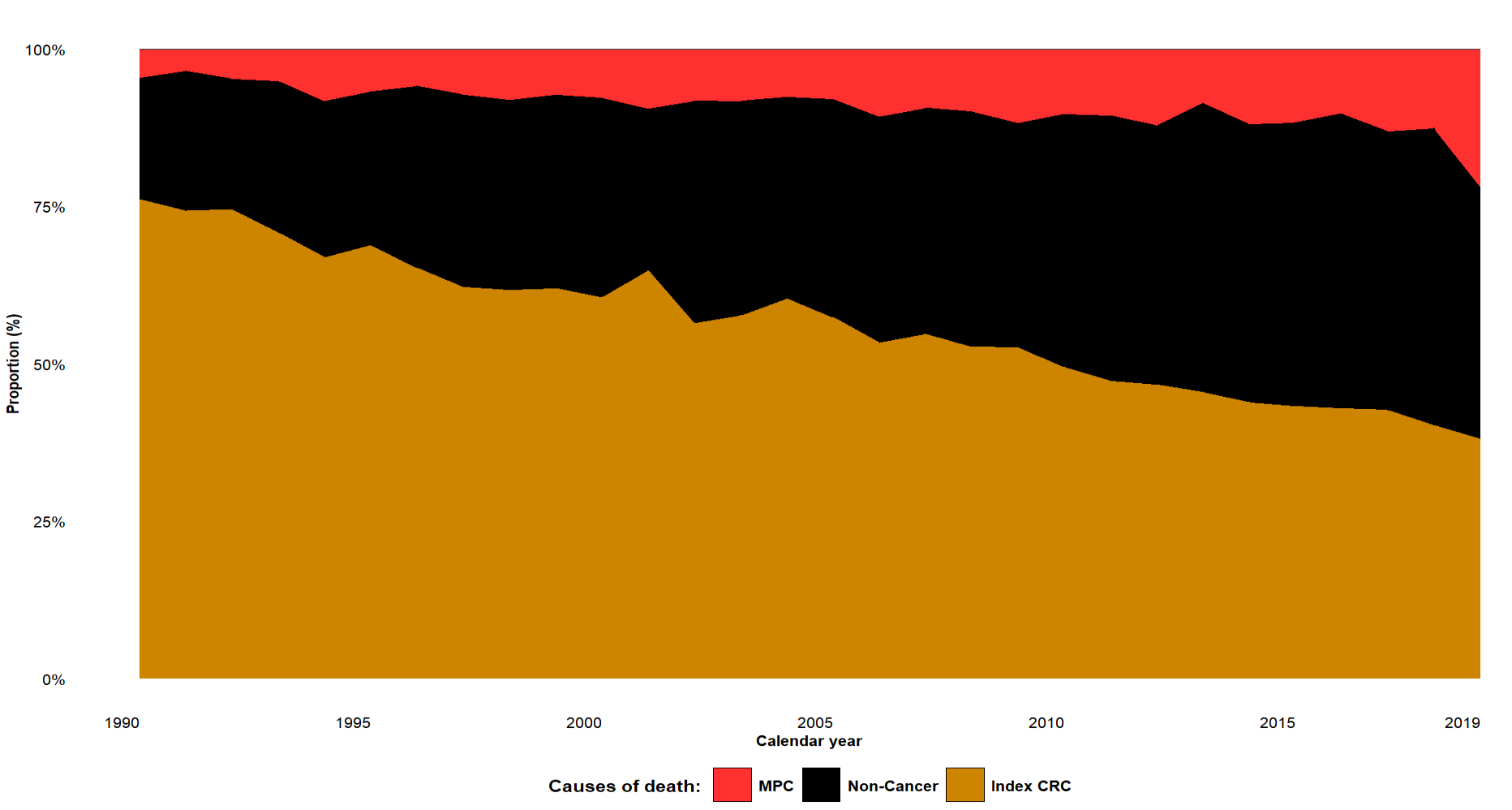


**Supplementary Figure 4**: Competing causes for all-cause mortality by calendar year in individuals diagnosed with CRC from 1990 to 2017.

CRC: colorectal cancer, MPC: multiple primary cancer


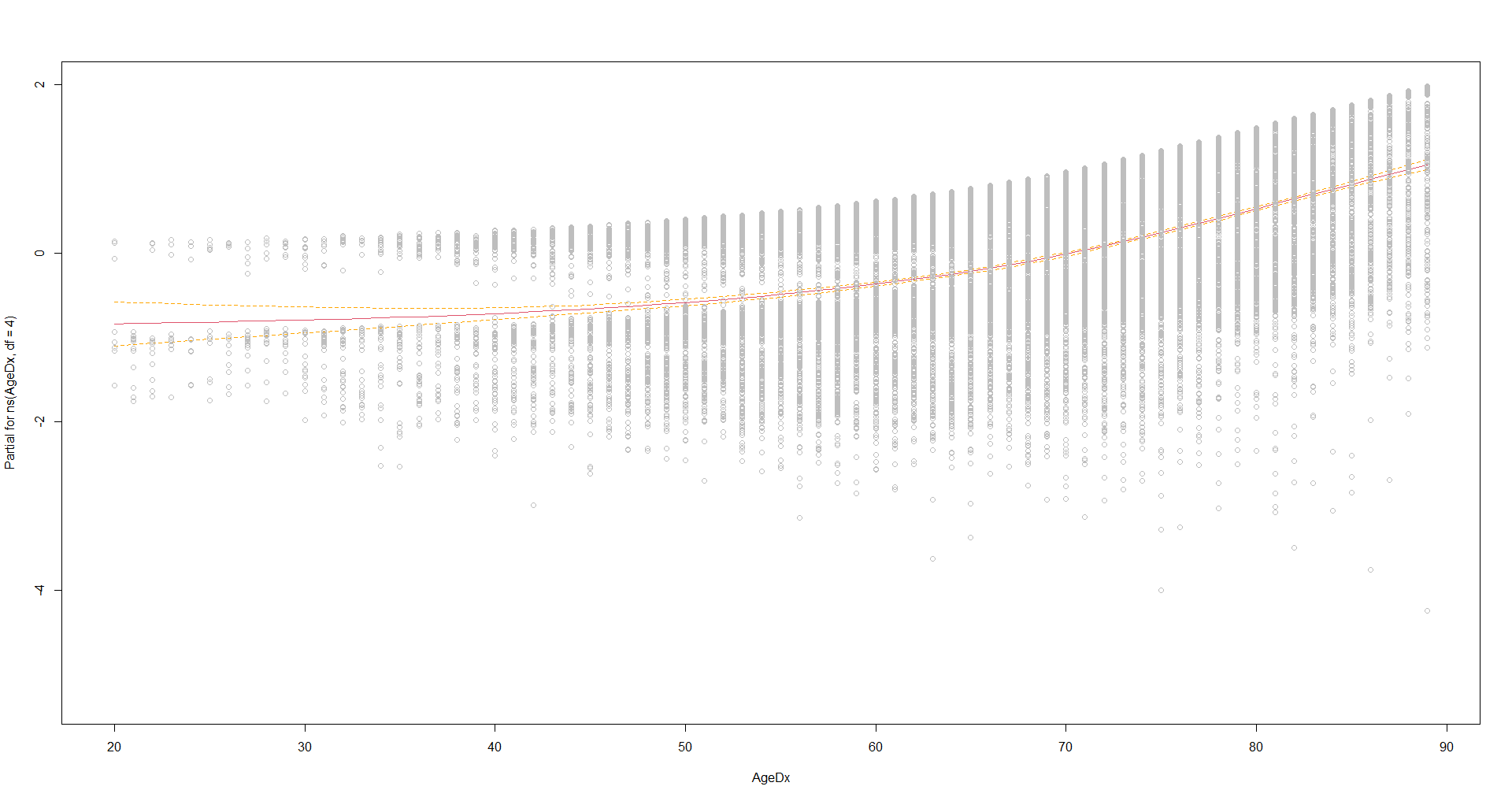


**Supplementary Figure 5:** The partial effect of the natural spline of age on the hazard of all-cause mortality among colorectal cancer survivors.

AgeDx: age the diagnosis of index CRC in years; df: degree of freedom; ns: natural spline.
